# Factors influencing stigma among healthcare professionals towards people who use illicit drugs in Japan: A quantitative study

**DOI:** 10.1002/pcn5.125

**Published:** 2023-08-04

**Authors:** Munenori Katayama, Kanna Sugiura, So Fujishiro, Jun Konishi, Ken Inada, Norihito Shirakawa, Toshihiko Matsumoto

**Affiliations:** ^1^ Mental Health and Welfare Center Yokohama Kanagawa Japan; ^2^ Department of Drug Dependence Research, Institute of Mental Health National Center of Neurology and Psychiatry Tokyo Japan; ^3^ Aichi Prefectural Mental Health and Welfare Center Nagoya Aichi Japan; ^4^ Department of Psychiatry, School of Medicine Kitasato University Sagamihara Kanagawa Japan

**Keywords:** drug, healthcare service, Japan, peer support, stigma

## Abstract

**Aim:**

Stigma among healthcare professionals toward people who use drugs (PWUDs) must be addressed for recovery. However, research on this topic is limited in Japan, therefore we developed a brand‐new scale through coproduction with PWUDs to measure stigma and conducted a survey using the developed scale to examine what influences stigma towards PWUDs in Japanese healthcare settings.

**Methods:**

Based on interviews with PWUDs and their families, we developed a survey containing 24 questions on stigma toward PWUDs. The survey was sent to healthcare professionals working in the public sector. Exploratory factor analysis (EFA) and confirmatory factor analysis (CFA) were conducted to determine the factor construct. Generalized linear mixed model (GLMM) analyses with each factor of the stigma questions set as a dependent variable were conducted to discover the specific contribution of each variable to professionals' stigma.

**Results:**

The six factors suggested by the EFA showed a good fit, as confirmed by the CFA of the stigma questions. GLMM discovered that “currently providing treatment services to PWUDs,” “having PWUDs close to themselves,” and “experiencing violence by the client when providing treatment services” were significantly associated with higher stigma scale scores. “Experience in receiving support,” “attending self‐help groups,” and “using peer‐based recovery support with PWUDs” were significantly associated with lower stigma scale scores.

**Conclusion:**

The scale coproduced with local PWUDs can be a reliable tool to measure the stigma PWUDs face in Japan. Further results indicate that interaction with recovered PWUDs should be promoted.

## INTRODUCTION

Stigma is defined as “a phenomenon that exists when elements of labeling, stereotyping, separating, status loss and discrimination co‐occur in a power situation that allows these processes to unfold.”^1^ People who use drugs (PWUDs) are known to face severe stigma compared to other psychiatric conditions.^2^, ^3^ People commonly hold the notion that PWUDs should be responsible for any consequences resulting from drug use.^4^, ^5^ Other common misconceptions include the idea that drug use is a moral problem^6^, ^7^ or that PWUDs lack willpower,^5^, ^7^ are dangerous ^5^ or are frightening.^5^, ^8^ When facing such prejudice, PWUDs decide to either hide their history of drug use or restrict their access to further healthcare services,^9^ either of which would result in social isolation and encourage further drug use.^6^, ^10^, ^11^ Even for PWUDs who do not need support, the existence of stigma is known to negatively impact their quality of life.^12^

### Drug use and stigma in Japan

As the world shifts from prohibition to regulation of illicit drugs, Japanese officials maintain their zero‐tolerance policy, treating illicit drugs as a solely criminal problem.^13^, ^14^ Schools teach how dangerous illicit drugs are and how they ruin lives under the slogan of *Dame‐Zettai* (Say “No, Absolutely No!” to Drug Abuse),^15^ which is a strategy abandoned in many nations years ago due to scientific research concluding its ineffectiveness.^16^

Officials say the policy deserves credit for reducing people's chances of exposure to illicit drugs,^17^ but drug treatment professionals criticized this as not only being ineffective but also worsening the stigma towards PWUDs.^14^, ^18^ These protests are backed by the fact that the number of people who use illicit drugs has not shown a meaningful decrease in recent years. In Japan, the lifetime experience rate for any illicit drug was 2.2% in 2017,^19^ 2.5% in 2019,^20^ and 2.4% in 2021,^21^ and the drug crime recidivism rate remains as high as 70.1%.^22^ The public label PWUDs as failures,^23^ not worthy of recognition or respect,^8^, ^18^ and perceive drug use as unacceptable.^8^, ^24^ PWUDs are unnoticed in the Japanese community as a result of this prejudice.^25^ In cases where their existence is recognized by the local community, PWUDs commonly experience forced segregation or discrimination^26^ caused by amplified fear. Moreover, a recent study found that companies hiring people with various disabilities were more hesitant to employ former PWUDs than people with other mental health conditions.^27^

Even professionals are not immune to such prejudice. Although the topic is hugely understudied in Japan, there are reports of a person being dismissed from their role as a probation officer once it was known that they had a family member who suffered from drug problems.^23^ Moreover, specialists argue that many emergency hospital and medical facility staff specialized in the care of PWUDs tend to recognize PWUDs as violent, dangerous, and not worthy of care despite the lack of evidence.^18^, ^28^ Hence, the problem of being reported to the police on hospital visits remains a major concern for PWUDs.

### Current research on healthcare professionals' stigma towards PWUDs

A systematic review^29^ discovered that healthcare professionals generally hold strongly negative attitudes towards PWUDs regardless of their field of specialty. They tend to label PWUDs as violent and disobedient, and responsible for their condition. Having a personal history of drug use, frequent contact with PWUDs, collaborative working environments and role support, knowledge about PWUDs, media portrayals, and culture are factors that may influence the professionals' stigma.^29^, ^30^

The results of studies aimed to address stigmatized attitudes of professionals were mixed.^31^ Social contact with the stigmatized group when training future clinicians is preferred for addressing stigma toward mental health conditions in general,^32^ but little is known on how the interaction should be in the case of stigma towards PWUDs.

Moreover, current data suffer from multiple limitations. One is the research setting, as most studies were conducted in Western nations. Considering the nonnegligible role of culture in forming stigma,^33^ further research among Asian nations, including Japan, is needed for a more accurate understanding of the phenomena, but a nationwide quantitative study does not exist in Japan, despite the high demand.

Considering the cultural aspects when modifying vignettes and scales to sufficiently cover stigma in each unique culture and satisfy content validity is crucial for accurate interpretation, especially in the case of stigma,^34^, ^35^, ^36^, ^37^, ^38^ as the phenomena are highly sensitive to each culture. Existing scales such as the Japanese version of the Reported and Intended Behaviour Scale^39^ and the Japanese version of King's Stigma Scale^40^ are not certain if they can measure stigma accurately in the Japanese context because the voices of Japanese people who suffer from mental illness were not taken into consideration during the scale's modification process. Another study that developed the Mental Illness and Disorder Understanding Scale^41^ was co‐produced with 30 people with mental illness in the scale development process, but the scale is designed to measure stigma towards mental illness in general and is not particularly adapted for PWUDs. Link's Perceived Discrimination‐Devaluation Scale^42^ is also capable of measuring perceived stigma towards people with various mental health issues. The scale is applied to numerous studies throughout the world but also did not incorporate the lived experiences of those who suffer from mental illness or drug use in the Japanese version.^43^ Here shows a need to develop a scale capable of accurately measuring stigma towards PWUDs in Japan.

### Study aim

To fill the current gap of knowledge around stigma towards PWUDs in Japan, the current study was conducted with the following purposes:

(1) To develop a scale solely measuring stigma toward PWUDs in Japan, in coproduction with local PWUDs.
(2) To conduct a nationwide survey to discover the effects of stigma toward PWUDs on public healthcare staff and its possible determinants.
(3) To establish the relationship between stigma and certain understudied phenomena which may influence stigma, such as the quality of interaction with PWUDs, as well as discovering specific factors correlating to certain stigma domains.

## METHODS

### Definition of PWUDs

We defined PWUDs as people who use(d) any sort of illicit drug. Substances such as alcohol, over‐the‐counter drugs, and prescribed drugs were excluded as these people were assumed to experience different stigma because these drugs are legal.

### Stigma questions

We used the Japanese version of the Link Stigma Scale as the basis for our scale, which consists of 12 questions pertaining to various aspects of perceived stigma. This scale was chosen since it was designed to control the social‐desirability bias by asking the respondent about the opinions of people around them instead of their own, rating each item on a four‐point Likert scale ranging from *strongly disagree* (1) to *strongly agree* (4). The level of stigma on the respondent is shown by calculating the total scale score. This structure was considered especially important since our target group was healthcare professionals and social desirability would hugely influence their response. On modification, the following process was used.

#### Review of current studies

Rather than conducting a review of preexisting international studies, we decided to cite two well‐designed systematic reviews published at the time of the study.^5^, ^38^ All articles referenced in the systematic review, including the study itself, were reviewed by the authors.

Additionally, we searched for domestic articles on the stigma towards PWUDs. A literature search was conducted on google scholar and Cinii under the search terms “薬物依存症” [drug addiction] AND “スティグマ” [stigma] OR “偏見” [prejudice] OR “差別” [discrimination]. As we were unable to find a peer‐reviewed study under the search terms, we decided to add magazine articles, short commentary papers, and unreviewed online articles to the search.

Each piece of literature was examined, and the findings were compared with the Link Stigma Scale. The researchers, all of whom had expertise working with PWUDs, proposed new items as candidates for the questionnaire whenever decided that none of the existing questions did not represent the reviewed study.

In the final process of this phase, multiple discussions were held by the authors to check the validity of each item as well as its wording. As a result, 22 items were retained as potential candidates for the study. Added items are marked with † in Table 1.

**Table 1:** List of the survey questions and its factor construct.

	Cronbach's *α* (total) = 0.89	Mean (SE)	Factor 1: Mistrust *α* = 0.75	Factor 2: Disregard *α* = 0.79	Factor 3: Devaluation *α* = 0.75	Factor 4: Denial *α* = 0.65	Factor 5: Discrimination *α* = 0.67	Factor 6: Dangerousness *α* = 0.86
2	Most people believe that a person who has used drugs in the past is just as intelligent as the average person*	2.32 (0.05)	0.57	−0.03	0.00	−0.15	−0.04	0.30
3	Most people believe that a former drug user is just as trustworthy as the average citizen*	2.71 (0.05)	0.98	−0.01	0.06	0.10	0.02	−0.08
18	Most people feel that a person can have a happy life even if they have used drugs in the past,†, *	2.14 (0.05)	0.03	0.59	0.15	−0.05	0.02	−0.10
22	Most people can understand the feeling of a person who used drugs,†, *	2.81 (0.05)	0.08	0.52	−0.10	0.20	0.03	0.00
23	Most people feel that a person who used drugs has only themselves to blame†	2.59 (0.05)	−0.06	0.44	0.14	0.11	0.11	0.23
24	Most people listen to what a person who has used drugs has to say,†, *	2.29 (0.05)	−0.02	0.93	−0.03	−0.13	0.07	0.03
5	Most people feel that using drugs is a sign of personal failure	2.44 (0.06)	0.10	−0.08	0.71	0.05	−0.12	0.07
6	Most people would not hire a person with a history of drug use to take care of their children, even if he or she had been well for some time	2.66 (0.05)	0.04	−0.07	0.60	0.06	0.20	−0.11
7	Most people think less of a person who has used drugs	2.40 (0.06)	−0.04	0.18	0.71	−0.06	−0.19	0.24
9	Most employers will pass over the application of a former drug user in favor of another applicant	2.88 (0.05)	−0.02	−0.01	0.52	0.02	0.22	−0.12
20	Most people think that there would not be anyone close to them with a history of drug use†	3.01 (0.05)	−0.11	−0.09	0.00	0.54	0.02	0.32
21	Most people think that they would never use drugs the way people with drug problems do†	3.27 (0.05)	0.11	−0.09	0.03	0.87	0.00	−0.09
10	Most people in my community would treat a person with a history of drug use just as they would treat anyone*	3.01 (0.04)	0.16	0.05	−0.18	0.03	0.50	−0.06
11	Most young people would be reluctant to date a person who has a history of using drugs	2.72 (0.05)	−0.06	−0.21	0.14	−0.13	0.43	0.14
13	Most people would not be willing to let their children play with a child of a person who has used drugs in the past, even if the person had been well for some time‡	2.64 (0.05)	0.02	0.09	0.04	0.04	0.62	0.13
14	Most people would not want to have relationships with neighbors who used drugs in the past‡	2.77 (0.05)	−0.06	0.14	−0.05	−0.01	0.68	0.33
17	Most people feel that people who used drugs in the past are dangerous†	2.78 (0.05)	0.00	0.09	0.06	0.01	0.33	0.66
19	Most people feel that people who used drugs are frightening†	2.92 (0.05)	0.00	0.13	0.00	0.00	0.37	0.54
1	Most people would willingly accept a person with a history of drug use as a close friend*	2.77 (0.04)	Deleted					
4	Most people would accept a fully recovered person with a history of drug use as a teacher of young children in a public school*	2.76 (0.05)	Deleted					
8	Most employers will hire a person with a history of drug use if he or she is qualified for the job*	2.52 (0.05)	Deleted					
12	Once they know a person has used drugs in the past, most people will take a person's opinions less seriously	2.49 (0.04)	Deleted					
15	Most people think that people who used drugs are morally wrong†	2.44 (0.06)	Deleted					
16	Most people feel that people who used drugs lack willpower†	2.55 (0.06)	Deleted					

Abbreviation: SE, standard error.

* Items which were reversely scored.

^†^ Items added from the literature review.

^‡^ Items added based on the interview.

#### Interview to peers

We conducted semistructured interviews with PWUDs and the families of PWUDs. This process was adopted (1) to learn about their experience of stigma in Japan to discover new survey questions that previous researchers may have missed and (2) to evaluate the content validity of the questions through cognitive interviewing.^38^ Families were included in the interview as well since this was a preliminary process of scale development. We aimed to gather as many episodes as possible from families as well since these episodes may not be recognized as a stigma by PWUDs themselves, but may be interrelated.^44^ PWUDs were contacted through a recovery facility for PWUDs and family members were contacted through a nonprofit organization supporting families of PWUDs. One author (M.K.) contacted the person in charge of each facility to recruit willing participants. Interviews were voluntary, individual, semistructured, and lasted about 1 hour. The typical interview began by asking the person the question, “What societal difficulties have you experienced recovering from drug problems?”, which was followed by a discussion on the subject. Rather than asking for direct episodes related to stigma, this question was presented to evoke the various difficulties experienced in the interviewee's social life, which may not be recognized as related to the stigmatization of drug use.

All interviews were audio‐recorded and transcribed. Initial analysis was carried out on each interview and the results were compared with the preexisting questions. New items were introduced whenever none of the preexisting questions reflected a participant's episode. Interviews were continued with snowball sampling until the following three criteria were met: (1) suggested minimum sample for reflexive thematic analysis^45^ (>6) was achieved, (2) no additional items were introduced from the latest interview, and (3) an equal number of interviews were conducted for PWUDs and families of PWUDs. After all three criteria were achieved, a final qualitative analysis was conducted for all interview data and the results were checked to ensure consistency with the set of developed questions.

Reflexive thematic analysis^46^ was conducted on the interview data to construct potential themes. This method was chosen because not only is it ideal for studies aimed at “‘giving voices’ to a socially marginalized group,”^47^ but it is also capable of capturing latent meaning in the data as our interview questions gathered episodes that may not have been recognized as episodes of stigma by the participant.^48^ The transcribed interviews were examined based on the concept of stigma presented above. All relevant data were coded and verified manually over multiple readings of the interviewee's statements. Afterward, each coded item was categorized based on the thematic framework. Coding and thematic analysis were carried out by one of the authors (M.K.) and reviewed by another (K.S.). Coding and categorization were conducted via Dedoose.^49^ All quotes were translated from Japanese to English and edited for clarity by the authors.

This process added two questions to the preexisting items. These questions are marked with ‡ in Table 1. Finally, a discussion was held by the researchers to reach a unanimous agreement when voting for the eligibility of each item for the scale.

### Participants of the survey

The set of stigma questions was sent to healthcare professionals in mental health and welfare centers (MHWCs), 69 local governmental facilities established in all 47 prefectures of Japan providing counseling and consultation services to the local population as stipulated by the Japanese law. Healthcare workers in MHWCs were asked to complete the survey either online or by e‐mailing the survey in Excel, Word, or pdf format.

### Demographic data collected in the survey

In addition to the set of stigma questions, the following were collected as independent variables: (a) gender, (b) age group, (c) currently providing treatment services to PWUDs, (d) has PWUDs close to themselves, (e) has met a recovered PWUD either in public or in private, (f) has/had a drug problem themselves, (g) has sought help for their own or their relative's drug problem, (h) has attended treatment programs for PWUDs (as participant, provider or visitor), (i) has attended self‐help groups for PWUDs (as participant or visitor), and (j) has heard recovery stories from recovered PWUDs. We did not define a “recovered PWUD” because the term recovery has various meanings for each individual and we thought that defining from the researcher's side would compromise the autonomy and the diversity of recovery for PWUDs. We therefore did not mention what “recovery” referred to but instead gave space for the respondent to choose based on their experience of interaction with PWUDs.

For respondents who were currently in the role of consulting PWUDs, the following questions about the quality of practice were also asked: (k) frequency, (l) year, (m) experience using peer‐based recovery support with PWUDs, (n) has experienced violence by the client when providing treatment services.

### Analysis

Exploratory factor analysis (EFA) and confirmatory factor analysis (CFA) were conducted on the stigma questionnaire to discover the factor construct. The suitability of data for EFA was examined using the Kaiser–Meyer–Olkin index (KMO) of sampling adequacy and Bartlett's *χ* ^2^ test of sphericity. The KMO index was compared with adequacy standards of 0.9, and *p* < 0.05 was set for Bartlett's *χ* ^2^ test of sphericity.^50^ EFA was performed using the maximum likelihood and the Promax rotation method because the factors were believed to be correlated. The number of factors was determined based on the suggested values from parallel analysis using squared multiple correlation. Minimum factor loading was set at <0.40.^50^

CFA was conducted based on the factor construct suggested by EFA. We used a combination of the comparative fit index (CFI), the Tucker–Lewis index (TLI), and the goodness fit index (GFI) to evaluate the fit index of the model. A model with a good fit is indicated by a number more significant than 0.9 for all fit indices.^51^

Internal consistency reliability was assessed by measuring Cronbach's *α* coefficient for each factor. The minimal value for Cronbach's *α* coefficient was set at 0.60.^50^

Finally, generalized linear mixed model (GLMM) analyses with significance tests were conducted with the total stigma scale score and each extracted stigma questionnaire factor as dependent variables. GLMM was chosen as it is preferred for achieving robust results considering the random effects in a regression model with mixed data of continuous and categorical variables.^52^, ^53^ First, analysis was conducted for all respondents, with questions (c) to (j) being set as the independent variables. Next, post hoc GLMM analyses were conducted based on results that showed a significant correlation to question (c) (currently providing treatment services to PWUDs) to examine the specific quality of practice influencing the stigma of professionals encountering PWUDs. For this, only the respondents who answered yes to question (c) were included, with questions (k) to (l) being set as the independent variables. Across all analyses, questions (a) and (b), and the type of survey the respondent used (either online form, Excel, Word, or pdf) were fitted as the random effect. The significance level was set at 0.05. All statistical analyses were performed via R.^54^ CFA was conducted with the “lavaan” package and GLMM with the “lme4” and “lmerTest” packages.

## RESULTS

### Interview

Four PWUDs and four family members were interviewed. All four PWUDs were male, had abstained from drug use for more than 10 years, and were employed. Three had one or more children. All four family members were parents of the PWUD (two fathers, and two mothers). Of the eight interviewees, two were using methamphetamine, two were using new psychoactive substances (kiken drugs^55^), and four were using multiple drugs including cannabis, new psychoactive substances, and methamphetamine. The mean duration of the interviews was 67 min and 23 s (standard deviation 15min and 23 s). Thematic analysis constructed eight distinctive themes (Table 2).

**Table 2:** Result of thematic analysis.

Theme	Description	Number of intercepts	Representative example
Cannot seek help	Many interviewees, regardless of their relationship with PWUDs, feel that seeking external help is a difficult task due to the negative image people hold of drug use.	43	“His brother suffered sleeping problems (due to being in the same room as a person using drugs)… All I could do was apologize, and tell him that I will do something, that I will somehow fix it (the drug problem) because no one else would help us.” (Family member)
			“I learned during my time in the recovery facility that I was supposed to be honest and open, but once I started working, I had to hide my condition. I felt quite exhausted at the time.” (PWUD)
Cannot be proud of themselves	Despite being abstinent for a long period, “recovered” PWUDs feel ashamed of themselves due to various stigma related to drug use.	29	“My kids can never be proud that their parent is working as a recovered counselor.” (PWUD)
			“I used to use methamphetamine. It's a crime, something that's against the law. That's something shameful as a working person.” (PWUD)
Internalizing prejudice	PWUD typically “swallow” people's stigmatized attitudes as they feel responsible for the troubles they caused when using drugs.	24	“I did really bad things. It (drug addiction) may be a medical condition, but it doesn't give you the right to steal. So, when people say harsh things about drug addicts, I feel that I deserve it.” (PWUD)
			“living with my fellows that understand my condition was really helpful. People who aren't interested in addiction might see us negatively, but I have to accept it.” (PWUD)
Criminality as a cause for stigmatized attitudes	People around did not overreact on disclosure of the history of drug use, as the drug was not illegal at the time.	31	“The drug wasn't illegal back then, so my relatives were like ‘Oh' and that was it. If it were methamphetamines or marijuana, they would have reacted totally differently.” (Family member)
			“I'm not able to be open about my condition to neighbors. Many people don't view us as someone with a disease.” (PWUD)
Lack of understanding	Caregivers and healthcare professionals lack the understanding that drug use is a medical condition. People thus criticize the actions of PWUDs even though the behavior may be part of the disorder.	35	“The caregiver scolded him in front of everyone. Criticizing that he didn't keep his promise. I mean, isn't it (drug addiction) a medical condition!?” (Family member)
			“I had to hide my condition when I was searching for a job. Even to job consultants. That was a quite difficult time for me.” (PWUD)
Being shown understanding and support	PWUDs as well as families felt that receiving support instead of stigmatized attitudes was a promoting factor for recovery from drug problems.	39	“The teacher said that he'll do as much as he can (to support my son, who was suffering from drug use). If it wasn't for him, we wouldn't have recovered.” (Family member)
			“Working as a recovered counselor really suits me. I can stay mentally healthy that I work with people who understands and supports me.” (PWUD)
Having to hide their condition	Even after long years of abstinence, PWUDs hesitate to disclose their condition to neighbors and other people around them, because such disclosure might result in labeling and bad attributes to not only the PWUD but their family as well, and negatively impact their relationship.	38	“It's something that I've done, so I don't mind what people say about me, but I'm always worried about my past causing problems to my kids.” (PWUD)
			“I was once ordered to make brochures promoting “*Dame‐Zettai*”, while I have a child who is suffering from drug addiction. I had to do it, I couldn't resist, but it was quite frustrating.” (Family member)
Restricted social activity	Fears of being labeled as bad cause PWUD and their families to hesitate from attending various social activities and connecting with neighbors.	36	“I still feel that I don't want to contact my son's classmates’ mothers.” (Family member)
			“I need to lie to people at social gatherings. You know, like times when you're afforded alcohol at parties. It makes me really nervous.” (PWUD)

Abbreviation: PWUDs, people who use drugs.

Theme 1 **Cannot seek help**

Many interviewees, regardless of their relationship with PWUDs, feel that seeking external help is a difficult task due to the negative image people hold of drug users.

His brother suffered sleeping problems (due to being in the same room as a person using drugs). All I could do was apologize and tell him that I will do something, that I will somehow fix it (the drug problem) because no one else would help us. (Family member)

Theme 2 **Cannot be proud of themselves**

Despite being abstinent for a long period, “recovered” PWUDs feel ashamed of themselves due to various stigma related to drug use.

My kids can never be proud that their parent is working as a recovered counselor. (PWUD)

Theme 3 **Internalizing prejudice**

PWUDs typically “swallow” people's stigmatized attitudes as they feel responsible for the troubles they caused when using drugs.

I did really bad things. It (drug addiction) may be a medical condition, but it doesn't give you the right to steal. So, when people say harsh things about drug addicts, I feel that I deserve it. (PWUD)

Theme 4 **Criminality as a cause for stigmatized attitudes**

People around did not overreact on disclosure of the history of drug use, as the drug was not illegal at the time.

The drug wasn't illegal back then, so my relatives were like “Oh” and that was it. If it were methamphetamines or marijuana, they would have reacted totally differently. (Family member)

Theme 5 **Lack of understanding**

Caregivers and healthcare professionals lack the understanding that drug use is a medical condition. People thus criticize the actions of PWUDs even though the behavior may be part of the disorder.

The caregiver scolded him in front of everyone. Criticizing that he didn't keep his promise. I mean, isn't it (drug addiction) a medical condition!? (Family member)

Theme 6 **Being shown understanding and support**

PWUDs as well as families felt that receiving support instead of stigmatized attitudes was a promoting factor for recovery from drug problems.

The teacher said that he'll do as much as he can (to support my son, who was suffering from drug use). If it wasn't for him, we wouldn't have recovered. (Family member)

Theme 7 Having to hide their condition

Even after long years of abstinence, PWUDs hesitate to disclose their condition to neighbors and other people around them because such disclosure might result in labeling and bad attributes not only for the PWUDs but their family as well, and negatively impact their relationship.

It's something that I've done, so I don't mind what people say about me, but I'm always worried about my past causing problems to my kids. (PWUD)

Theme 8 **Restricted social activity**

Fears of being labeled as bad cause PWUDs and their families to hesitate attending various social activities and connecting with neighbors.

I still feel that I don't want to contact my son's classmates' mothers. (Family member)

In addition to the interview, all eight interviewees confirmed that the set of questions was understandable and adequately reflected the interviewees' perceived stigma.

### Survey

Two hundred and twenty‐nine people responded (response rate 66.4%). Seven responses were excluded due to missing variables. Two hundred and twenty‐two responses were included for analysis. Of the 222 respondents, 75.7% were female and 88.7% were currently in the practice of consulting PWUDs (Table 3). Ceiling effects or floor effects were not observed in any of the stigma scale questions (Table 1).

**Table 3:** Demographic variables of the survey respondents.

Description	*N* (%/standard error)
(a) Gender	
Male	53 (23.9%)
Female	168 (75.7%)
Other	1 (0.05%)
(b) Age group	
20s	23 (10.4%)
30s	53 (23.9%)
40s	89 (40.1%)
50s	42 (18.9%)
60s	15 (6.8%)
70s	0 (0.0%)
(c) Currently providing treatment services to PWUD	
Yes: No	197 (88.7%):25 (11.3%)
(d) Has a PWUD close to them	
Yes: No	27 (12.2%):195 (87.8%)
(e) Has met a recovered PWUD either in public or private	
Yes: No	195 (87.8%):27 (12.2%)
(f) Has/had drug problem him/herself	
Yes: No	3 (1.4%):219 (98.6%)
(g) Has sought help for their own or a relative's drug problem	
Yes: No	8 (3.6%):214 (96.4%)
(h) Has attended treatment programs for PWUDs	
Yes: No	134 (60.4%):88 (39.6%)
(i) Has attended self‐help groups for PWUDs	
Yes: No	140 (63.1%):82 (36.9%)
(j) Had heard stories of recovery from a recovered PWUD	
Yes: No	67 (30.2%):155 (69.8%)
(k) Frequency of consulting people with drug problems	
Everyday	7 (3.6%)
Once per week or more	23 (11.7%)
Once per month or more	74 (37.6%)
Once per year or more	69 (35%)
Less than once per year	24 (12.2%)
(l) Mean year of experience (standard error)	4.84 (5.25)
(m) Experience using peer‐based recovery support with PWUDs	
Yes: No	107 (54.3%):90 (45.7%)
(n) Has experienced violence by the client when providing treatment services	
Yes: No	31 (15.7%):166 (84.3%)

Abbreviation: PWUDs, people who use drugs.

### Factor construct

Results from the KMO index (0.93) and Bartlett's *χ* ^2^ test of sphericity (*P* < 0.01) indicated that the data were adequate for EFA. Parallel analysis indicated the number of factors should be six. Five items were excluded on initial EFA, as these items did not show a good fit for any of the factors. One item was also excluded due to double loading. EFA was re‐run, and all items were sufficiently loaded to one of the factors (Table 1).

CFA showed that the fit indices for the model suggested by EFA were all within the acceptable limits (CFI = 0.953, TLI = 0.939, GFI = 0.911).

### Internal consistency

Cronbach's *α* coefficient for the total scale was 0.89. Coefficients for factors 1 to 6 were 0.75, 0.79, 0.75, 0.65, 0.67, and 0.86, respectively. All values were above the suggested minimum limit (Table 1).

### The naming of the six factors

Each factor was labeled below based on its factor construct for better interpretation of further analyses^56^ (Table 1).

Factor 1 (items 2, 3): Mistrust
Factor 2 (items 18, 22, 23, 24): Disregard
Factor 3 (items 5, 6, 7, 9): Devaluation
Factor 4 (items 20, 21): Denial
Factor 5 (items 10, 11, 13, 14): Discrimination
Factor 6 (items 17, 19): Dangerousness

### GLMM

Tables 4 and 5 detail the full results of the GLMMs examining relationships between the stigma questionnaire score and each independent variable. “Currently providing treatment services to PWUD” was significantly associated with higher stigma for the total stigma score (coefficient = 5.30, standard error = 1.70, *T* = 3.12, *P* value = 0.01), and the Disregard (coefficient = 1.41, standard error = 0.52, *T* = 2.72, *P* value = 0.01), Devaluation (coefficient = 1.30, standard error = 0.54, *T* = 2.40, *P* value = 0.02), Discrimination (coefficient = 1.34, standard error = 0.41, *T* = 3.27, *P* value = 0.00), and Dangerousness (coefficient = 0.82, standard error = 0.30, *T* = 2.74, *P* value = 0.01) factors. Having a close PWUD was also significantly associated with higher stigma for the Mistrust factor (coefficient = 0.54, standard error = 0.26, *T* = 2.05, *P* value = 0.04). On the other hand, having received support for their own or a relative's drug problem was significantly associated with lower stigma for the total stigma score (coefficient = −5.47, standard error = 2.77, *T* = −1.98, *P* value = 0.05), and the Discrimination (coefficient = −1.39, standard error = 0.67, *T* = −2.08, *P* value = 0.04) and Dangerousness (coefficient = −0.96, standard error = 0.49, *T* = −1.97, *P* value = 0.05) factors. Attending self‐help groups was also significantly associated with lower stigma for the total stigma score (coefficient = −2.43, standard error = 1.16, *T* = −2.10, *P* value = 0.04) and the Discrimination factor (coefficient = −0.61, standard error = 0.28, *T* = −2.15, *P* value = 0.03). Other independent variables did not have a significant association with any of the factor scores.

**Table 4:** Results of generalized linear mixed model analysis (for all respondents).

		(c) Currently providing treatment services to PWUDs			(d) Has a PWUD close to them			(e) Has met a recovered PWUD either in public or in private			(f) Has/had drug problems him/herself			(g) Sought for help for their own or a relative's drug problem			(h) Attended treatment programs for PWUDs			(i) Attended self‐help groups for PWUDs			(j) Heard stories of recovery from a recovered PWUD		
*N* = 222	Mean (SE)	Coef (SE)	*T*	*P*	Coef (SE)	*T*	*P*	Coef (SE)	*T*	*P*	Coef (SE)	*T*	*P*	Coef (SE)	*T*	*P*	Coef (SE)	*T*	*P*	Coef (SE)	*T*	*P*	Coef (SE)	*T*	*P*
Total	8.35 (0.53)	5.30 (1.70)	3.12	0.01**	2.47 (1.60)	1.55	0.12	−1.28 (3.14)	−.41	0.71	−2.90 (4.49)	−.65	0.52	−5.47 (2.77)	−1.98	0.05*	.15 (1.32)	0.12	0.91	−2.43 (1.16)	−2.10	0.04*	.12 (1.94)	0.07	.95
Factor 1: Mistrust	5.02 (0.09)	0.36 (0.28)	1.31	0.19	0.54 (0.26)	2.05	0.04*	−0.48 (0.46)	−1.06	0.38	−0.54 (0.74)	−0.72	0.47	−0.85 (0.63)	−1.35	0.46	0.25 (0.19)	1.27	0.2	−0.15 (0.19)	−0.78	0.44	−0.03 (0.21)	−0.14	0.89
Factor 2: Disregard	9.84 (0.16)	1.41 (0.52)	2.72	0.01*	0.74 (0.5)	1.47	0.14	−0.27 (0.8)	−0.33	0.75	−0.41 (1.38)	−0.3	0.77	−1.5 (0.86)	−1.74	0.08	0.39 (0.36)	1.07	0.29	−0.59 (0.46)	−1.27	0.29	0.09 (0.4)	0.23	0.82
Factor 3: Devaluation	10.38 (0.16)	1.3 (0.54)	2.4	0.02*	0.72 (0.52)	1.39	0.17	0.2 (0.73)	0.28	0.79	−1.74 (1.45)	−1.2	0.23	−0.36 (0.9)	−0.4	0.69	−0.66 (0.43)	−1.55	0.17	−0.22 (0.38)	−0.59	0.55	0.29 (0.45)	0.65	0.56
Factor 4: Denial	6.28 (0.08)	0.26 (0.27)	.95	0.35	0.13 (0.25)	.52	0.61	−0.29 (0.43)	−0.68	0.53	0.73 (0.72)	1.01	0.31	0.28 (0.97)	0.29	0.82	0.22 (0.19)	1.15	0.25	−0.36 (0.19)	−1.94	0.05	0.08 (0.29)	0.27	0.81
Factor 5: Discrimination	11.13 (0.13)	1.34 (0.41)	3.27	0.00**	0.01 (0.39)	.02	0.98	−0.17 (0.71)	−0.24	0.82	−0.62 ((1.09)	−0.57	0.57	−1.39 (0.67)	−2.08	0.04*	−0.02 (0.3)	−0.07	0.95	−0.61 (0.28)	−2.15	0.03*	0 (0.53)	0	1
Factor 6: Dangerousness	5.70 (0.09)	0.82 (0.3)	2.74	0.01*	0.35 (0.28)	1.25	0.21	−0.05 (0.51)	−0.09	0.93	−0.6 (0.79)	−0.77	0.44	−0.96 (0.49)	−1.97	0.04*	−0.16 (0.21)	−0.76	0.45	−0.2 (0.23)	−0.86	0.44	−0.1 (0.3)	−0.33	0.76

Abbreviation: Coef, coefficient; PWUDs, people who use drugs; SE, standard error.

* *P* < 0.05

** *P* < 0.01.

**Table 5:** Results of generalized linear mixed model analysis (for respondents currently providing treatment services to PWUDs).

		(k) Frequency of practice			(l) Year of practice			(m) Experience using peer‐based recovery support with PWUDs			(n) Has experienced violence by the client when providing treatment services		
*N* = 197	Mean (SE)	Coef (SE)	*T*	*P*	Coef (SE)	*T*	*P*	Coef (SE)	*T*	*P*	Coef (SE)	*T*	*P*
Total	48.97 (0.56)	−0.07 (0.64)	−0.12	0.90	0.02 (0.11)	0.14	0.89	−2.73 (1.25)	−2.18	0.03*	5.46 (2.68)	0.14	0.89
Factor 2: Disregard	9.99 (0.17)	−0.11 (0.20)	−0.58	0.56	0.05 (0.04)	1.26	0.26	−0.51 (0.39)	−1.31	0.19	1.07 (0.53)	2.02	0.09
Factor 3: Devaluation	10.52 (0.18)	0.01 (0.20)	−0.04	0.97	0.03 (0.04)	0.65	0.55	−0.98 (0.40)	−2.45	0.01*	1.07 (0.83)	1.28	0.29
Factor 5: Discrimination	11.28 (0.13)	−0.01 (0.15)	0.05	0.96	−0.03 (0.03)	−1.03	0.31	−0.29 (0.30)	−0.98	0.33	1.13 (0.41)	2.75	0.01*
Factor 6: Dangerousness	5.80 (0.10)	0.01 (0.11)	0.06	0.96	−0.01 (0.02)	−0.76	0.45	−0.45 (0.21)	−2.11	0.04*	0.79 (0.38)	2.08	0.12

Abbreviation: Coef, coefficient; PWUDs, people who use drugs; SE, standard error.

* *P* < 0.05.

Among post hoc analyses of respondents who currently provide treatment services to PWUDs, experiencing violence by the client when providing treatment services was significantly associated with higher stigma for the Discrimination factor (coefficient = 1.13, standard error = 0.41, *T* = 2.75, *P* value = 0.01). Experience using peer‐based recovery support with PWUDs was significantly associated with lower stigma for the total stigma score (coefficient = −2.73, standard error = 1.25, *T* = −2.18, *P* value = 0.03), and the Devaluation (coefficient = −0.98, standard error = 0.40, *T* = −2.45, *P* value = 0.01) and Dangerousness (coefficient = −0.45, standard error = 0.21, *T* = −2.11, *P* value = 0.04) factors. Other independent variables did not have a significant association with any of the factors.

## DISCUSSION

This first Japanese study to collaborate with PWUDs and their families in addition to applying rigid statistical analysis was successful in building a scale measuring stigma towards PWUDs in the Japanese context. We discovered that Japanese healthcare professionals who currently support PWUDs in public facilities, and especially those who were exposed to hostile behavior, held a higher level of stigma compared to those who were unexposed. Conversely, having received support on a relative's drug problems, attending self‐help groups, and collaborating with recovered PWUDs upon clinical practice associated with lower stigma.

Although results were mostly consistent with those of other nations, we were surprised to discover that clinical practices were associated with increased stigma among Japanese healthcare professionals, which was contradictory to studies elsewhere. We emphasize that this study was able to discover the prefered style of interaction with PWUDs to decrease stigma.

Considering the importance of clinical education for stigma reduction, lack of training opportunities may be causing stigma among professionals who encounter PWUDs in clinical practice as the current curriculum for healthcare professionals in Japan seldom mentions this topic.^57^ We suggest that training programs should recommend collaborative practice with recovered PWUDs, making visits to self‐help groups, as well as suggesting clinicians seek help if they carry unresolved trauma with someone's drug problems. Consistent with the definition of stigma presented by Link, these measures can correct the power imbalance between PWUDs and healthcare providers. These measures are essential to prevent the stigmatization of PWUDs, especially in the Japanese context, since many students will have gone through the stigma‐promoting *Dame‐Zettai* school curriculum during their early education years. These school programs tend to portray PWUDs in a very negative light, but witnessing the massive impact of how recovered PWUDs can contribute to clinical practice^58^ may bring a paradigm shift so that clinicians start to see PWUDs as people worthy of respect.

A supplementary, but important finding of this study was that the proportion of professionals who experienced violence during the clinical practice with PWUDs was as low as 15.7%. This does not mean that these experiences can be ignored. Emphasis should be put on protecting clinicians from becoming victims, as well as providing aftercare if violence occurs. Our findings, however, imply that many generally held beliefs about PWUDs in Japan may be false and that being a PWUD will not automatically render a person aggressive.

We believe applying this newly developed scale to future studies can contribute to a better understanding of stigma towards PWUDs in Japan and develop concrete interventions. Not only has the scale been localized to reflect the particular experience of PWUDs in Japan, but we also discovered specific domains of stigma in our scale. This is especially important as seen from our analysis that each factor influenced different domains. Application of this scale can prevent interventions from being misinterpreted as null results where they were successful in reducing certain stigma domains. Moreover, studies could be conducted in other settings to discover what structural differences or institutional policies are influencing stigma towards PWUDs in Japanese healthcare settings.

### Limitations

The study exhibits several limitations. First, the development of the scale may have encountered selection bias, as only male PWUDs and parents of PWUDs were interviewed. The experiences of female PWUDs, other family members including children, spouses, or siblings, and PWUDs who have not maintained abstinence may entail distinct forms of stigma. Additionally, certain articles were overlooked during the literature review as the process was not conducted systematically. We also report that the survey results cannot be generalized as only healthcare staff in public sectors were recruited. Moreover, the voluntary nature of the survey may have introduced information bias as individuals with particularly negative attitudes towards PWUDs may have chosen not to participate, leading to potential favorable response bias. Furthermore, due to the self‐reporting format of the survey, some degree of information bias may persist, as participants may have provided favorable responses regarding their demographic variables, given that the survey was administered to healthcare providers. Confounding bias may also exist since certain demographic variables, such as the respondents' qualifications or history of training, were not collected. Finally, the analytical approach employed for the interviews may have influenced the scale development process. Additional items could be introduced when using alternative methodologies.

## CONCLUSION

Stigma has been recognized as a significant factor affecting the experiences of PWUDs, and its implications may be crucial. This pioneering nationwide quantitative survey, conducted in collaboration with PWUDs and their families, explored possible determinants of Japanese public healthcare professionals' stigma towards PWUDs using a newly developed stigma scale specifically designed for this purpose. Our findings also imply that promoting the inclusion and active participation of PWUDs as co‐producers across various aspects of clinical practice and drug‐related issues may serve as an effective strategy to address and mitigate stigma. Aligned with the principle of “nothing about us without us,” addressing power imbalances is crucial in combating stigma, and fostering the role of peers as a valuable resource to meet the needs of both clinicians and PWUDs can empower both parties and contribute to the reduction of stigma within the community.

## AUTHOR CONTRIBUTIONS

Munenori Katayama designed the study, participated in the analysis, and led the manuscript writing. Kanna Sugiura participated in the qualitative analysis and manuscript revision. Sou Fujishiro designed the study, organized the survey recruits, and participated in manuscript revision. Jun Konishi participated in the quantitative analysis and manuscript revision. Ken Inada participated in manuscript revision. Norihito Shirakawa participated in designing the study and organized the survey recruits. Toshihiko Matsumoto provided the funding and participated in manuscript revision. All authors have read and approved the final manuscript.

## CONFLICT OF INTEREST STATEMENT

The authors declare no conflict of interest.

## ETHICS APPROVAL STATEMENT

This study was approved by the Zenkoku Seishin Hoken Fukushi Center Head Committee (Head Committee of Japan's Mental Health and Welfare Center).

## PATIENT CONSENT STATEMENT

Informed consent was obtained from all participants in the study in written form.

## CLINICAL TRIAL REGISTRATION

n/a

## Data Availability

Data used in this study can be accessed through correspondence with the lead author.
